# The Self-Perpetuating Cycle of Psychological Distress and Clinical Outcomes in Head and Neck Oncology

**DOI:** 10.3390/cancers18091491

**Published:** 2026-05-06

**Authors:** Ankita Chakrawal, Aashruti Pathania, Ruhi Dixit, Manoj Pandey

**Affiliations:** 1 HTAIn Resource Centre, Institute of Medical Sciences, Banaras Hindu University, Varanasi 221005, India; ankitachakrawal1991@gmail.com (A.C.); ruhi.dixit3@bhu.ac.in (R.D.); 2 Department of Surgical Oncology, Institute of Medical Sciences, Banaras Hindu University, Varanasi 221005, India; aashruti.pathania@gmail.com

**Keywords:** distress, anxiety, depression, quality of life, survival

## Abstract

**Background:** Head and neck cancer (HNC) presents unique challenges due to impairments in vital functions and appearance. This narrative synthesis of systematic review synthesizes evidence on the prevalence and predictors of distress in HNC and proposes a Multifactorial Cycle of Distress to explain how psychological burdens compromise clinical outcomes. **Methods:** A systematic search of PubMed and Scopus was conducted (up to June 2025). From 342 articles, 32 were included, covering HNC patients with quantitative or qualitative distress assessments related to quality of life (QoL) and survival. **Results:** Distress prevalence ranged from 23–47%, with 30% of survivors experiencing persistent symptoms long-term. Key predictors included advanced tumor stage, radiotherapy-induced side effects, and pre-treatment depression, which increased distress risk 2- to 3-fold. Notably, distress was associated with a 25% reduction in radiotherapy completion and a 55% increased risk of mortality. Additionally, 71% of patients experienced decision regret, further impacting outcomes. Psychosocial interventions, including cognitive-behavioral therapy and mindfulness, showed potential in reducing anxiety by 15–20%. **Conclusions:** Distress in HNC functions as a self-perpetuating cycle where physical morbidity triggers psychological barriers that directly impair treatment adherence and survival. Routine screening using tools like the Distress Thermometer or DIC-2 and integrated multidisciplinary care are essential to break this cycle and improve patient and caregiver well-being.

## Simple Summary

This article explores the self-perpetuating cycle of psychological distress in head and neck oncology, illustrating how functional impairments and physical disfigurement trigger a cascade of negative clinical outcomes. Synthesizing data from 32 studies, the review reports a distress prevalence of 23–47%, which is associated with a 25% reduction in radiotherapy completion and a 55% increase in mortality risk. The authors introduce a “Multifactorial Model of the Distress-Outcome Cycle” to map the recursive biological and behavioral feedback loops that compromise survival. Ultimately, the study advocates for routine distress screening and integrated multidisciplinary care as essential strategies to break this cycle and improve the quality of life for both patients and their caregivers.

## 1. Introduction

Head and neck cancer (HNC) remains one of the most challenging malignancies in modern oncology, accounting for over 550,000 new cases globally every year. This burden is set against a broader oncological landscape where approximately 20 million new cancer cases are diagnosed annually, resulting in 9.7 million deaths [1]. With the global cancer burden projected to rise by 77% by the year 2050, the physical and psychosocial challenges associated with these diseases are expected to escalate proportionately. Within this context, HNC is distinct due to its profound impact on the most fundamental aspects of human identity and survival, including speech, swallowing, breathing, and physical appearance.

The management of HNC typically involves intensive multimodal treatments such as surgery, radiotherapy, and chemotherapy. While these interventions are essential for curative intent, they frequently result in significant functional impairments and visible disfigurement [2,3]. These physical changes trigger a cascade of psychological distress, manifesting as clinical anxiety, depression, a persistent fear of cancer recurrence (FCR), and debilitating body image concerns [2,3,4]. This distress is further exacerbated by socioeconomic factors, particularly in low-resource settings, where financial insecurity and a lack of social support create additional barriers to patient well-being.

Crucially, the impact of psychological distress in HNC extends far beyond emotional suffering; it is a critical determinant of clinical success. Evidence suggests that pretreatment depression is associated with a 25% reduction in radiotherapy completion rates, largely mediated by increased pain and fatigue [5]. This lack of adherence, combined with the biological effects of chronic stress, such as the activation of the hypothalamic-pituitary-adrenal (HPA) axis and the resulting suppression of immune function, leads to significantly poorer outcomes [5,6,7]. Indeed, patients with depression face a 55% increased risk of mortality compared to those without it [8].

While the correlation between psychological distress and quality of life is well-documented, a critical gap persists in the current literature regarding the lack of an integrated, HNC-specific framework that maps the recursive biological and social feedback loops. Existing models often fail to account for the unique “social death” and functional isolation inherent in head and neck oncology, where the loss of vital communicative and swallowing mechanisms initiates a distinct cascade of decline. This review addresses this void by proposing a “Multifactorial Model of the Distress-Outcome Cycle,” [9] moving beyond general psycho-oncological narratives to explore how cultural stigmas, such as those observed in the Asia-Pacific region where gender norms discourage seeking support, further exacerbate social withdrawal [10].

Furthermore, the introduction of spirituality and religiosity as coping mechanisms probably represents a burgeoning frontier in HNC care. Recent scoping reviews suggest that for many patients, religious faith provides a crucial buffer against existential distress and improves pain management, yet these factors are rarely integrated into standard clinical outcome models [11]. By synthesizing these diverse psychosocial variables, from spiritual resilience to social determinants of health, this review provides a proposed roadmap for identifying early “interruption points” in the distress cycle.

## 2. Methods

### 2.1. Search Strategy and Study Identification

This narrative synthesis was conducted by performing a systematic search of the PubMed and Scopus databases for peer-reviewed literature published up to June 2025 following the Preferred Reporting Items for Systematic Reviews and Meta-Analyses (PRISMA) guidelines to ensure a transparent and reproducible selection process, PRISMA checklist please see Supplementary File S2. The search strategy utilized a combination of Boolean operators (OR) and targeted keywords, including “head and neck cancer,” “distress,” “anxiety,” “depression,” “quality of life,” “treatment compliance,” and “psychosocial interventions”. The detailed strategy is reported in Supplementary File S1. This initial identification phase yielded a total of 342 articles, comprising 198 records from PubMed and 144 from Scopus.

### 2.2. Eligibility Criteria and Selection Process

**Figure 1:**
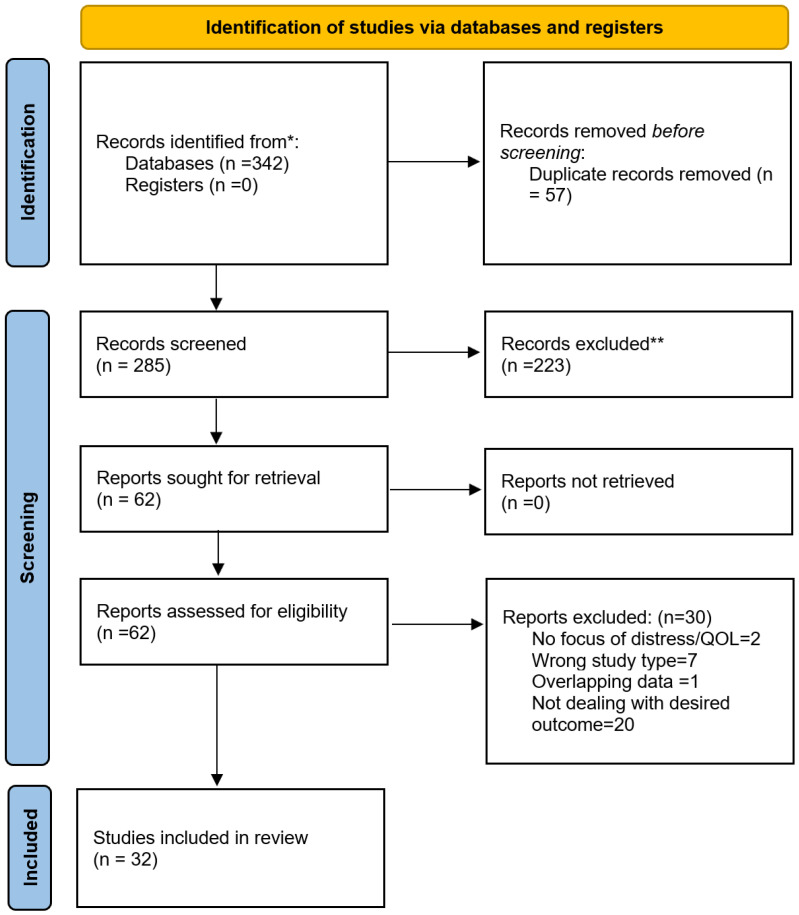
PRISMA 2020 flow diagram of the study selection process. The flowchart illustrates the systematic identification, screening, and inclusion of literature for this narrative review.

Following the removal of 57 duplicates, 285 articles were screened by title and abstract. Studies were eligible for inclusion if they involved HNC patients across any stage or treatment modality, utilized quantitative or qualitative distress assessments, and reported outcomes related to QoL, treatment adherence, or survival. Exclusion criteria were applied to studies focusing on non-HNC cancers, those lacking distress-related outcomes, and non-English publications. After a detailed full-text review of 62 candidate articles, a final cohort of 32 studies including systematic reviews, cohort studies, and intervention trials was selected for inclusion (Figure 1).

### 2.3. Data Extraction and Narrative Synthesis

Data were systematically extracted from the included studies, focusing on study design, sample size, and specific distress measures such as the Hospital Anxiety and Depression Scale (HADS), Distress Thermometer, and Distress Inventory for Cancer (DIC-2). Additionally, findings related to QoL tools (e.g., EORTC QLQ-C30, FACT-HN), treatment compliance, and clinical outcomes were meticulously recorded. Given the significant heterogeneity in study designs and outcome measures across the literature, neither a meta-analysis nor a formal quantitative quality assessment (e.g., Newcastle-Ottawa Scale) was performed. The narrative synthesis was employed to organize and present the evidence according to thematic domains including prevalence, predictors, impact on quality of life, treatment compliance, and clinical impacts.

### 2.4. Narrative Synthesis and Model Development

Due to the significant heterogeneity in study designs, a narrative synthesis approach was used to categorize findings into four domains: prevalence of distress, multi-dimensional predictors, impact on treatment compliance, and clinical outcomes. These findings were then integrated to develop the proposed conceptual Multifactorial Model of the Distress-Outcome Cycle.

## 3. Results

### 3.1. Prevalence of Distress

The prevalence rates for distress ranged from 23–47% across HNC patient populations [9,12,13,14,15]. A systematic review of patients receiving radiotherapy found that 30% experienced clinically significant anxiety and 26% reported depression during treatment that decreased over a period of 6 months following treatment [4,16]. Another study of 123 HNC patients undergoing multimodal treatment reported a mean distress score of 24.6 on the Distress Inventory for Cancer (DIC2), with 40% exceeding the clinical threshold for distress [17]. Long-term survivors (7–11 years post-treatment) showed persistent distress in 30% of cases, particularly those with cognitive or social impairments [18,19]. Caregivers also reported high distress, with FCR levels comparable to or exceeding those of patients [20,21].

### 3.2. Predictors of Distress

**Figure 2:**
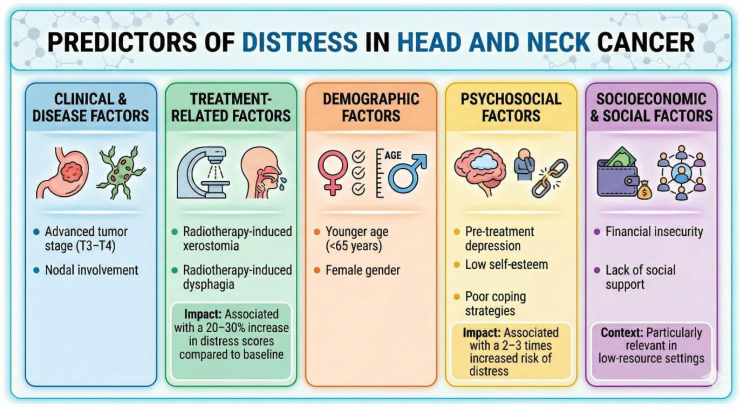
Multidimensional predictors and risk factors for distress in HNC. This infographic categorizes the clinical, treatment-related, demographic, and psychosocial variables that contribute to patient distress. Clinical factors include advanced tumor stage (T3–T4) and nodal involvement. Treatment-related morbidity, specifically radiotherapy-induced xerostomia and dysphagia. Psychosocial predictors, such as pre-treatment depression and poor coping strategies, correlate with a 2- to 3-fold increased risk of clinically significant distress. Socioeconomic barriers, including financial insecurity and lack of social support, further exacerbate this burden, particularly in low-resource settings.

The studies looking at the predictors identified numerous variables contributing to distress. Advanced tumor stage (T3–T4) and nodal involvement were consistently associated with higher distress levels [22]. Treatment-related factors, such as radiotherapy-induced xerostomia and dysphagia, increased distress scores by 20–30% compared to baseline [23,24,25]. Younger patients (<65 years) reported higher distress in physical domains, while females exhibited greater emotional distress [3,26]. Pre-treatment depression, low self-esteem, and poor coping strategies were significant predictors, increasing distress risk by 2–3 times [27]. Financial insecurity and lack of social support were reported in 10 studies as exacerbating distress, particularly in low-resource settings (Figure 2).

### 3.3. Impact on Quality of Life

**Figure 3:**
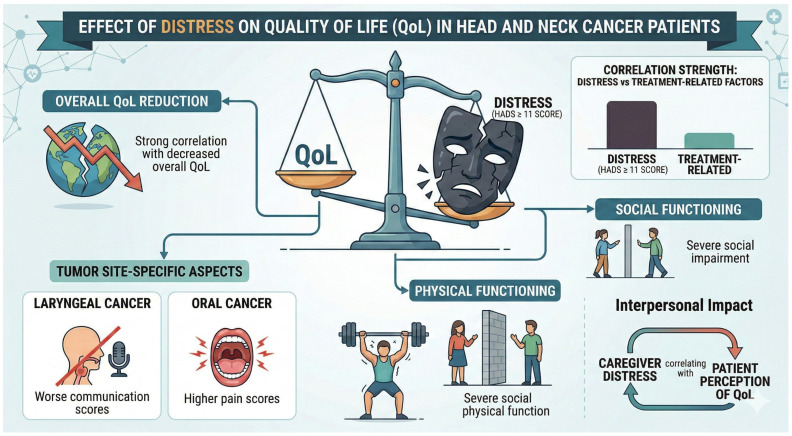
Impact of distress on Health-Related Quality of Life (HR-QoL): Higher distress levels (Hospital Anxiety and Depression Scale [HADS] ≥ 11) were associated with significantly poorer physical functioning (mean score 62 vs. 78, *p* < 0.01) and social functioning (mean score 55 vs. 72, *p* < 0.05) compared to patients with lower distress. Strong negative correlations were observed between distress scores and overall HR-QoL (r = −0.62 to −0.78). Site-specific differences are highlighted, including greater communication difficulties in laryngeal cancer and higher pain scores in oral cavity cancer. Caregiver distress and fear of cancer recurrence further negatively influenced patient-reported QoL. Data synthesized from multiple studies.

The studies assessing QoL primarily employed the EORTC QLQ-C30, QLQ-H&N35, and FACT-HN. Higher distress was strongly correlated with poorer QoL (r = −0.62 to –0.78 across studies) [4,17,28,29,30]. A cohort study of HNC survivors found that patients with HADS scores ≥ 11 reported significantly worse physical functioning (mean score: 62 vs. 78, *p* < 0.01) and social functioning (mean score: 55 vs. 72, *p* < 0.05) compared to those with lower distress [5]. Tumor site influenced QoL, with laryngeal cancer patients reporting worse communication scores and oral cancer patients experiencing higher pain scores. Advanced tumor stage was associated with poorer scores across QoL domains [19]. Caregiver distress also negatively impacted patient QoL perceptions, with caregiver depression correlating with a lower patient-reported QoL score (Figure 3).

The visual model depicts a negative correlation (r = −0.62 to −0.78) between high distress and patient well-being. High distress scores (HADS ≥ 11) are associated with significantly lower physical (62 vs. 78, *p* < 0.01) and social functioning (55 vs. 72, *p* < 0.05). The figure also highlights site-specific impairments, such as compromised communication in laryngeal cancer and increased pain scores in oral cancer. Additionally, it illustrates the interpersonal impact, where caregiver distress and fear of cancer recurrence (FCR) negatively influence the patient’s own perception of their quality of life.

### 3.4. Impact on Treatment Compliance

On examining the treatment compliance, focusing on surgery, radiotherapy and chemotherapy, pretreatment depression was found to be associated with a 25% reduction in radiotherapy completion rates [5]. Higher distress and emotional stress lead to significant treatment delay affecting the patient’s outcome [31]. Pain, fatigue, and financial barriers were key mediators of non-compliance, with distressed patients reporting a 30% higher symptom burden.

### 3.5. Impact on Clinical Outcomes

Distress has been linked to clinical outcomes [32]. An analysis of patients reported a 55% increased mortality risk (HR = 1.55; 95% CI = 1.36, 1.76) in patients with depression, compared to those without it [8], that is reduced by treatment of anxiety and depression [33]. Distress was also associated with higher rates of recurrence detection delays, potentially due to emotional disengagement. FCR was prevalent in 60–70% of patients and caregivers, correlating with increased anxiety and reduced QoL. Pooled prevalence of decision regret in studies was 71%, and together with decision conflict can influence clinical outcomes [34].

### 3.6. The Distress Cycle

**Figure 4:**
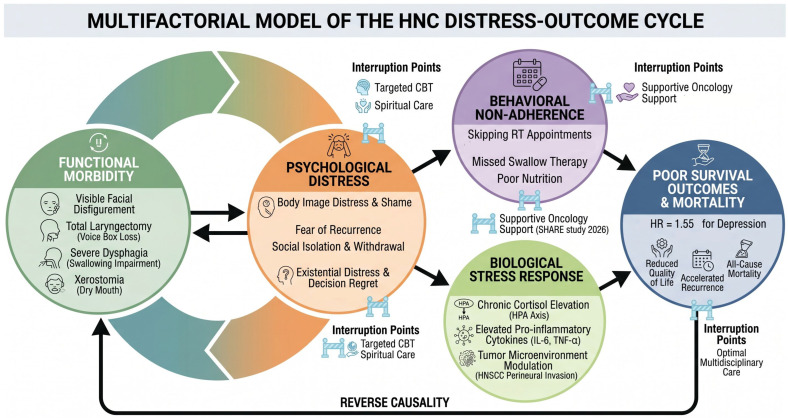
Multifactorial Model of the Head and Neck Cancer (HNC) Distress-Outcome Cycle. This schematic conceptualizes the recursive pathways linking initial functional morbidity to poor survival outcomes and mortality. The cycle is propelled by psychological distress, which drives both behavioral non-adherence and a biological stress response, culminating in reduced quality of life, accelerated recurrence, and mortality. The model highlights specific, evidence-based “Interruption Points” where targeted clinical interventions can mitigate these pathways and improve patient outcomes, breaking the self-perpetuating cycle.

The proposed distress cycle in head and neck cancer represents a complex, self-perpetuating feedback loop where the physical and psychological burdens of the disease continuously reinforce one another (Figure 4). The cycle begins with a cancer diagnosis and the subsequent functional impairments such as difficulties with speech, swallowing, and breathing, alongside visible disfigurement from surgery or radiotherapy [35]. These physical challenges trigger profound psychological distress, manifesting as anxiety, depression, and a persistent fear of cancer recurrence. This heightened distress often leads to behavioral changes, including reduced treatment adherence and emotional disengagement, which can result in a 25% reduction in radiotherapy completion rates [32]. Consequently, poor compliance and the biological effects of chronic stress such as immune suppression, compromise clinical outcomes, leading to higher recurrence risks and increased mortality. These worsening clinical outcomes then feed back into the cycle, further exacerbating the patient’s distress and diminishing their overall quality of life.

This integrated model proposes a self-perpetuating feedback loop between cancer-related burdens and patient outcomes. The cycle is initiated by a cancer diagnosis and subsequent functional impairments (e.g., dysphagia, disfigurement). These physical challenges trigger profound psychological distress, which directly influences clinical decision-making and treatment adherence. Evidence shows that distress results in a 25% reduction in radiotherapy completion. Reduced adherence, coupled with immune modification, leads to poorer clinical outcomes, including increased recurrence and mortality, which then feedback to exacerbate existing distress, further diminishing quality of life.

### 3.7. Interventions

Cognitive-behavioral therapy (CBT) and mindfulness-based therapy reduced anxiety by 15–20% in small trials but lacked large-scale RCTs for confirmation [18]. Among 133 patients evaluated using the Washington QoL instrument, patients undergoing guided imagery (GIFT) during radiotherapy showed a 10% reduction in distress scores (*p* = 0.04) [36]; however, an older Cochrane systematic review failed to show any benefit of psychological intervention on the QoL of head neck cancer patients in a review of 7 trials totaling 542 patients [18]. Multidisciplinary programs combining psychological support and symptom management improved QoL on the FACT-HN scale and introduction of a screening program helps [13]. Distress screening using the Distress Thermometer identified 40% of at-risk patients, with 75% accepting referrals to psychological support [5]. Caregiver-targeted interventions showed mixed results, with limited efficacy in reducing FCR.

## 4. Discussion

Distress is a pervasive issue in HNC, driven by the disease’s impact on critical functions such as speech, swallowing, and breathing, as well as its effects on appearance. The prevalence of distress, ranging from 23% to 47%, is significantly higher than in other cancers, and its persistence in long-term survivors underscores the need for ongoing psychological support [4,16]. Caregiver distress, particularly fear of cancer recurrence (FCR), amplifies the emotional burden, highlighting the importance of family-centered care [20,21]. Predictors of distress, including advanced tumor stage, treatment side effects, and psychosocial factors like poor coping strategies, suggest the need for tailored interventions [22,27]. Financial insecurity, especially in low-resource settings, further exacerbates distress, emphasizing the importance of addressing socioeconomic barriers.

The impact of distress on QoL is profound, with stronger correlations than treatment-related factors [4,5,28,29,30]. Tumor site-specific impairments, such as communication difficulties in laryngeal cancer, necessitate targeted rehabilitation [19]. Caregiver distress complicates QoL, as discrepancies between patient and caregiver perceptions can hinder support. Distress also reduces treatment adherence, particularly for radiotherapy, due to its intensive schedule and side effects, with pain and fatigue as key mediators. Interventions improving self-efficacy and symptom management show promise but are underutilized due to access barriers and stigma [5].

**Figure 5:**
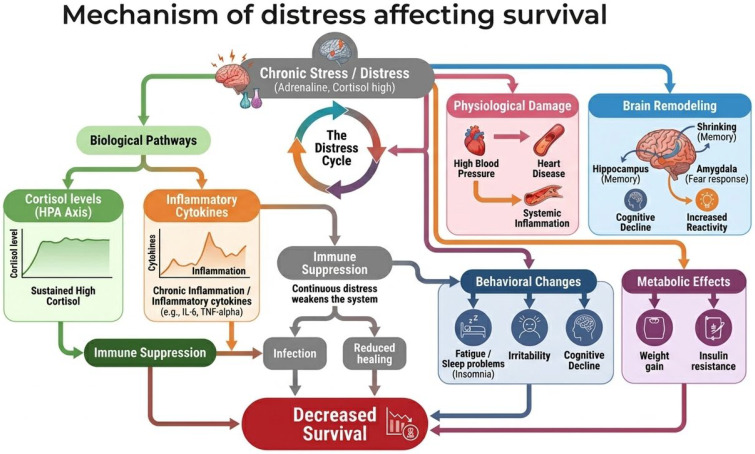
Biological mechanisms of survival: The impact of chronic stress. This diagram outlines the physiological pathways through which chronic distress compromises clinical outcomes. Prolonged activation of the Hypothalamic-Pituitary-Adrenal (HPA) axis leads to sustained high cortisol levels, which can suppress immune function. The resulting imbalance in inflammatory cytokines (e.g., IL-6, TNF-α) creates a pro-inflammatory environment that may promote tumor recurrence. These biological changes, combined with behavioral shifts such as fatigue and cognitive decline, collectively contribute to an increased mortality risk and poorer overall prognosis.

The link between distress and worse survival highlights the need to address psychological health as part of cancer care [8]. Chronic stress may suppress immune function, and distress-related non-compliance likely contributes to poorer outcomes (Figure 5). Fear of cancer recurrence remains a critical but underexplored factor affecting both patients and caregivers [20,21,37]. Interventions such as cognitive-behavioral therapy, mindfulness, and guided imagery are promising but require larger trials to establish efficacy [18]. Multidisciplinary programs combining psychological and symptom management improve QoL and compliance, while distress screening identifies at-risk patients for early intervention [5]. Caregiver-targeted interventions need further development to address fear of cancer recurrence effectively.

### 4.1. Clinical Relevance

**Figure 6:**
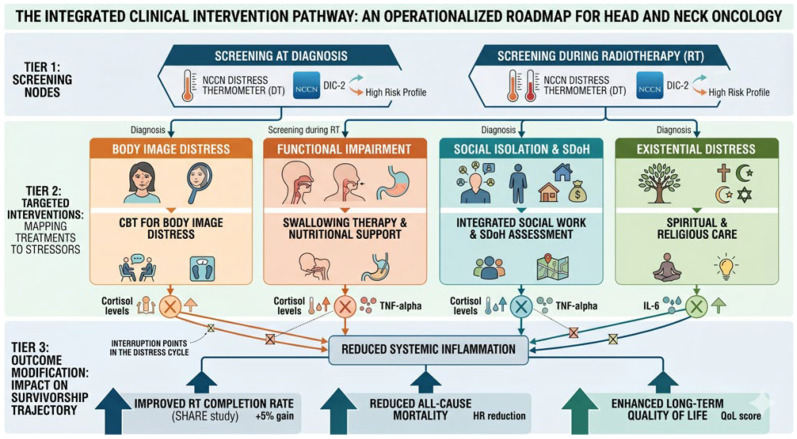
The Integrated Clinical Intervention Pathway. An operationalized roadmap for multimodal head and neck oncology care designed to break the self-perpetuating distress cycle. Tier 1 identifies critical screening nodes at diagnosis and during radiotherapy using validated tools like the NCCN Distress Thermometer and DIC-2. Tier 2 maps targeted, evidence-based interventions—including cognitive-behavioral therapy for body image distress, swallowing therapy and nutritional support, and spiritual care to specific patient stressors. Tier 3 demonstrates the resulting outcome modifications, such as improved radiotherapy completion rates, reduced all-cause mortality, and enhanced long-term quality of life.

This review underscores the critical need to integrate psychological care into routine HNC management, as untreated distress not only diminishes patient QoL and caregiver support but also compromises treatment adherence and survival. Clinicians should prioritize routine distress screening at diagnosis, during treatment, and in survivorship to identify at-risk individuals early as recommended in the guidelines, potentially improving survival and QoL. Tailored multidisciplinary interventions, including cognitive-behavioral therapy, mindfulness, and family-centered psychosocial support, offer practical avenues to mitigate fear of cancer recurrence and enhance outcomes, particularly in vulnerable groups. By fostering holistic care models, this approach can reduce mortality risks, alleviate socioeconomic barriers, and promote equitable access to mental health resources in oncology settings. The integrated clinical intervention pathway is detailed in Figure 6.

### 4.2. Confounding, Bias, and Social Determinants

A rigorous analysis of current evidence shows inherent inferential challenges in observational oncology data. The documented association between psychological depression and a 55% increased mortality risk requires interpretation within a broader framework of confounding variables [38]. Advanced disease stages (T3–T4) and treatment-induced morbidities such as severe dysphagia and xerostomia, act as powerful independent drivers of both psychological decline and mortality, often creating a “reverse causality” effect where distress serves as a prodromal marker of aggressive biological phenotypes rather than its primary cause. Furthermore, the distress cycle is frequently anchored in structural social determinants of health (SDoH). Recent multivariable analyses indicate that for every increase in adverse individual- and neighborhood-level factors, physical quality of life (QoL) decreases significantly, while depression severity rises [39]. These findings suggest that structural deprivation and income status are foundational predictors of sustained psychological morbidity 12 months post-treatment, independent of clinical disease stage [40].

### 4.3. Biological Specificity: The HNC Microenvironment

**Figure 7:**
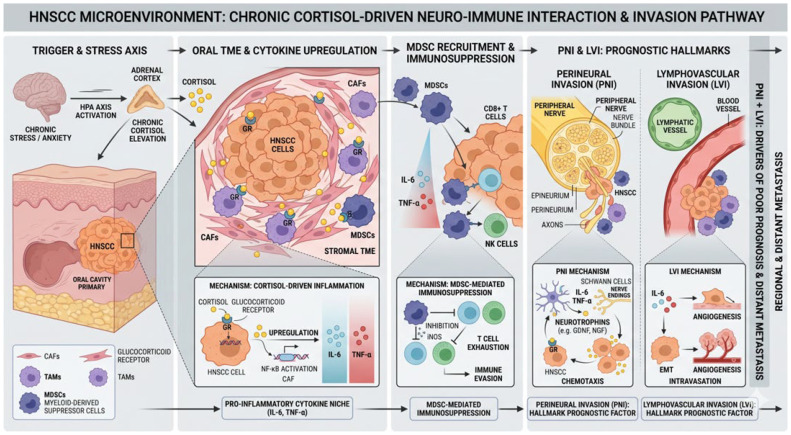
Neuro-Immune Interactions in the HNSCC Microenvironment. This diagram illustrates the physiological pathways through which chronic psychological distress and anxiety compromise clinical prognosis. Chronic activation of the hypothalamic-pituitary-adrenal (HPA) axis leads to sustained cortisol elevation, which promotes a pro-inflammatory niche in the tumor microenvironment (TME) characterized by the upregulation of cytokines such as IL-6 and TNF-α. This environment facilitates the recruitment of myeloid-derived suppressor cells (MDSCs) and systemic immune evasion. These biological changes directly contribute to hallmark prognostic factors, including perineural invasion (PNI) and lymphovascular invasion (LVI), which drive regional recurrence and distant metastasis.

The biological mechanisms driving the distress cycle in HNC involve a specific interplay between the hypothalamic-pituitary-adrenal (HPA) axis and the tumor microenvironment. Chronic activation of the HPA axis leads to sustained cortisol levels that create a pro-inflammatory environment characterized by an imbalance of cytokines such as IL-6 and TNF-α [41,42]. In the context of squamous cell carcinoma, this inflammatory cascade has been linked to increased perineural invasion and accelerated recurrence rates. This biological suppression, coupled with stress-induced “brain remodeling”, where chronic fear of recurrence may lead to amygdala hyperactivity and subsequent cognitive decline, forms a physiological core that reinforces the behavioral disengagement from intensive treatment schedules (Figure 7).

### 4.4. Measurement Heterogeneity and the Diagnostic Frontier

The “Distress Cycle” is further complicated by the heterogeneity of measurement tools employed across the literature. While the National Comprehensive Cancer Network (NCCN) Distress Thermometer is the clinical standard, traditional thresholds may fail to capture a significant proportion of HNC patients with meaningful psychosocial and physical concerns [43]. High-prevalence problems such as pain, eating changes, and worry often persist regardless of the reported “distress score,” highlighting the need for universal symptom assessment [43]. Consequently, identifying high-risk biopsychosocial “typologies” such as clusters characterized by high psychological burden and advanced disease, is essential for optimizing recovery trajectories and preventing the long-term persistence of distress seen in up to 30% of survivors [44].

### 4.5. The Intervention Landscape: Moving Toward RCT Evidence

The previous reliance on older systematic reviews is now being superseded by high-quality randomized controlled trials (RCTs). New evidence from the SHARE study demonstrates that structured psycho-oncology support integrated directly into radiotherapy schedules can significantly reduce the “adherence gap” previously observed in distressed populations [45]. Similarly, targeted cognitive-behavioral interventions like the BRIGHT program (2024) have shown efficacy in reducing the social isolation and body image shame specific to HNC-related disfigurement [46]. These programs emphasize individual psychological adjustment and trust-building as cornerstones of behavioral change [47]. The inclusion of spiritual care is also gaining recognition; spirituality and religiosity are frequently associated with enhanced coping capacity and emotional resilience, potentially serving as a buffer within the multifactorial cycle of distress [11].

### 4.6. Long-Term Survivorship and Work Disability

The impact of the distress cycle remains evident in late survivorship, specifically regarding socio-economic reintegration. Long-term work disability (LTD) is prevalent in approximately 33% of working-age HNC survivors more than five years post-treatment [48]. This disability is best explained by current body image distress and cognitive functioning rather than historical clinical stage, underscoring the persistence of the psychological feedback loop [48]. Even among young adults (18–45 years), clinically relevant distress is reported by over 50% of survivors, with returning to work identified as a key moderator that correlates with reduced anxiety and improved global QoL [49].

### 4.7. Critical Assessment of the Proposed Model

The proposed “Multifactorial Model of the Distress-Outcome Cycle” provides a necessary clinical roadmap for identifying specific interruption points in the feedback loop between functional impairment and survival. Its primary strength is the HNC-specific focus, integrating unique stressors like the loss of vital speech and swallowing mechanisms as catalysts for psychological and biological decline. However, it is important to acknowledge that the model currently serves as an analytical framework rather than a validated predictive tool; longitudinal, large-scale studies are required to precisely operationalize the statistical mediators between distress severity and long-term mortality. Nevertheless, aligning this model with the latest ESMO 2024 guidelines for communication and support in chronic cancer care provides a standardized path for enhancing patient-centric oncology [50].

### 4.8. Limitations

We acknowledge several methodological limitations inherent to the included studies and the synthesis process, including small sample sizes in many studies that restricted generalizability and statistical power; heterogeneous outcome measures, such as varying distress and QoL tools like HADS, Distress Thermometer, DIC-2, and EORTC QLQ-C30 that complicated direct comparisons across studies; and limited longitudinal data in some analyses that hindered tracking of distress trajectories over time. The majority of included studies utilized observational designs, which are inherently susceptible to confounding and issues of reverse causality. It remains difficult to determine whether psychological distress independently drives mortality or if advanced tumor stage and treatment-induced morbidities such as severe dysphagia, simultaneously escalate both distress levels and clinical decline. Consequently, the reported hazard ratios should be interpreted as strong associations rather than definitive causal links. Additionally, cultural and geographic variations in distress and QoL assessments were underexplored, with potential underrepresentation of diverse populations and low-resource settings, and no meta-analysis was feasible due to these inconsistencies, relying instead on narrative synthesis, which may introduce subjectivity.

## 5. Conclusions

This systematic review and narrative synthesis underscores that psychological distress in head and neck oncology is not a secondary symptom but a core clinical variable that operates within a complex, self-perpetuating cycle. Evidence indicates that high levels of anxiety and depression are associated with significantly lower treatment adherence and poorer survival outcomes, though these relationships are modulated by disease stage and functional impairment.

The proposed Multifactorial Model of the Distress-Outcome Cycle highlights that breaking this feedback loop requires more than just emotional support; it necessitates an integrated approach that addresses functional rehabilitation, social determinants of health, and biological inflammatory markers. Clinicians should move beyond generic screening to utilize biopsychosocial typologies that identify the most vulnerable patients early in the treatment trajectory. As randomized controlled trials (RCTs) begin to demonstrate the efficacy of integrated psycho-oncology support, it is imperative that multidisciplinary HNC teams incorporate standardized, evidence-based interventions as a primary component of curative intent care.

## Data Availability

No new data were created or analyzed in this study.

## Supplementary Materials

The following supporting information can be downloaded at https://www.mdpi.com/article/10.3390/cancers18091491/s1: File S1: search strategy. File S2: Prisma checklist
