# Supplementary material for: The Self-Perpetuating Cycle of Psychological Distress and Clinical Outcomes in Head and Neck Oncology
Source: Cancers (Basel). 2026 May 6;18(9):1491. doi: 10.3390/cancers18091491 (PMC13162879; doi:10.3390/cancers18091491)
Supplement: Supplementary file 1 [file cancers-18-01491-s001.zip › File S1. search strategy suppl.pdf]

Supplementary File S1: Search stretegy

Pubmed

(("Head and Neck Neoplasms"[MeSH Terms] OR "head and neck cancer"[Title/Abstract] OR "head and neck neoplasm*"[Title/Abstract] OR "head-neck cancer"[Title/Abstract] OR "HNC"[Title/Abstract] OR "oral cancer"[Title/Abstract]) AND ("Psychological Distress"[MeSH Terms] OR "distress"[Title/Abstract] OR "emotional distress"[Title/Abstract] OR "anxiety"[Title/Abstract] OR "depression"[Title/Abstract] OR "fear of cancer recurrence"[Title/Abstract] OR "FCR"[Title/Abstract] OR "body image"[Title/Abstract] OR "quality of life"[Title/Abstract] OR "QoL"[Title/Abstract] OR "psychosocial"[Title/Abstract] OR "psychological"[Title/Abstract] OR "mental health"[Title/Abstract]) AND ("survival"[Title/Abstract] OR "mortality"[Title/Abstract] OR "treatment adherence"[Title/Abstract] OR "compliance"[Title/Abstract] OR "radiotherapy completion"[Title/Abstract] OR "treatment completion"[Title/Abstract] OR "patient outcome*"[Title/Abstract] OR "clinical outcome*"[Title/Abstract] OR "disease recurrence"[Title/Abstract] OR "prognosis"[Title/Abstract])) AND ((clinicaltrial[Filter] OR meta-analysis[Filter] OR observationalstudy[Filter] OR randomizedcontrolledtrial[Filter] OR systematicreview[Filter]) AND (english[Filter]))

Scopus

((INDEXTERMS("Head and Neck Neoplasms") OR TITLE-ABS("head and neck cancer") OR TITLE-ABS("head and neck neoplasm*") OR TITLE-ABS("head-neck cancer") OR TITLE-ABS(HNC) OR TITLE-ABS("oral cancer")) AND (INDEXTERMS("Psychological Distress") OR TITLE-ABS(distress) OR TITLE-ABS("emotional distress") OR TITLE-ABS(anxiety) OR TITLE-ABS(depression) OR TITLE-ABS("fear of cancer recurrence") OR TITLE-ABS(FCR) OR TITLE-ABS("body image") OR TITLE-ABS("quality of life") OR TITLE-ABS(QoL) OR TITLE-ABS(psychosocial) OR TITLE-ABS(psychological) OR TITLE-ABS("mental health")) AND (TITLE-ABS(survival) OR TITLE-ABS(mortality) OR TITLE-ABS("treatment adherence") OR TITLE-ABS(compliance) OR TITLE-ABS("radiotherapy completion") OR TITLE-ABS("treatment completion") OR TITLE-ABS("patient outcome*") OR TITLE-ABS("clinical outcome*") OR TITLE-ABS("disease recurrence") OR TITLE-ABS(prognosis))) AND ((clinicaltrial[Filter] OR meta-analysis[Filter] OR observationalstudy[Filter] OR randomizedcontrolledtrial[Filter] OR systematicreview[Filter]) AND (english[Filter]
